# Fully automatic cardiac four chamber and great vessel segmentation on CT pulmonary angiography using deep learning

**DOI:** 10.3389/fcvm.2022.983859

**Published:** 2022-09-26

**Authors:** Michael J. Sharkey, Jonathan C. Taylor, Samer Alabed, Krit Dwivedi, Kavitasagary Karunasaagarar, Christopher S. Johns, Smitha Rajaram, Pankaj Garg, Dheyaa Alkhanfar, Peter Metherall, Declan P. O'Regan, Rob J. van der Geest, Robin Condliffe, David G. Kiely, Michail Mamalakis, Andrew J. Swift

**Affiliations:** ^1^Department of Infection, Immunity and Cardiovascular Disease, University of Sheffield, Sheffield, United Kingdom; ^2^3D Imaging Lab, Sheffield Teaching Hospitals NHSFT, Sheffield, United Kingdom; ^3^Insigneo Institute for in Silico Medicine, University of Sheffield, Sheffield, United Kingdom; ^4^Radiology Department, Sheffield Teaching Hospitals NHSFT, Sheffield, United Kingdom; ^5^Norwich Medical School, University of East Anglia, Norwich, United Kingdom; ^6^MRC London Institute of Medical Sciences, Imperial College London, London, United Kingdom; ^7^Department of Radiology, Leiden University Medical Center, Leiden, Netherlands; ^8^Sheffield Pulmonary Vascular Disease Unit, Sheffield Teaching Hospitals NHS Trust, Sheffield, United Kingdom; ^9^Department of Computer Science, University of Sheffield, Sheffield, United Kingdom

**Keywords:** deep-learning (DL), semantic segmentation and labelling, computed tomography pulmonary angiography (CTPA), whole heart segmentation, pulmonary vascular disease (PVD)

## Abstract

**Introduction:**

Computed tomography pulmonary angiography (CTPA) is an essential test in the work-up of suspected pulmonary vascular disease including pulmonary hypertension and pulmonary embolism. Cardiac and great vessel assessments on CTPA are based on visual assessment and manual measurements which are known to have poor reproducibility. The primary aim of this study was to develop an automated whole heart segmentation (four chamber and great vessels) model for CTPA.

**Methods:**

A nine structure semantic segmentation model of the heart and great vessels was developed using 200 patients (80/20/100 training/validation/internal testing) with testing in 20 external patients. Ground truth segmentations were performed by consultant cardiothoracic radiologists. Failure analysis was conducted in 1,333 patients with mixed pulmonary vascular disease. Segmentation was achieved using deep learning *via* a convolutional neural network. Volumetric imaging biomarkers were correlated with invasive haemodynamics in the test cohort.

**Results:**

Dice similarity coefficients (DSC) for segmented structures were in the range 0.58–0.93 for both the internal and external test cohorts. The left and right ventricle myocardium segmentations had lower DSC of 0.83 and 0.58 respectively while all other structures had DSC >0.89 in the internal test cohort and >0.87 in the external test cohort. Interobserver comparison found that the left and right ventricle myocardium segmentations showed the most variation between observers: mean DSC (range) of 0.795 (0.785–0.801) and 0.520 (0.482–0.542) respectively. Right ventricle myocardial volume had strong correlation with mean pulmonary artery pressure (Spearman's correlation coefficient = 0.7). The volume of segmented cardiac structures by deep learning had higher or equivalent correlation with invasive haemodynamics than by manual segmentations. The model demonstrated good generalisability to different vendors and hospitals with similar performance in the external test cohort. The failure rates in mixed pulmonary vascular disease were low (<3.9%) indicating good generalisability of the model to different diseases.

**Conclusion:**

Fully automated segmentation of the four cardiac chambers and great vessels has been achieved in CTPA with high accuracy and low rates of failure. DL volumetric biomarkers can potentially improve CTPA cardiac assessment and invasive haemodynamic prediction.

## Introduction

Pulmonary vascular disease encompasses a range of conditions that are linked with a large disease burden worldwide and are associated with high mortality and morbidity (1–4). Computed tomography pulmonary angiography (CTPA) is a crucial imaging investigation performed in patients with suspected pulmonary embolism (PE) and in the work up of patients with suspected pulmonary hypertension (PH) (1). Current imaging approaches in pulmonary vascular disease rely on visual assessments or manual measurements of cardiac, pulmonary arterial and aortic size; such measures are used to risk stratify patients with acute PE (5–8) and diagnose PH (5, 6).

Pulmonary arterial dilatation is a salient feature radiologists observe on routine thoracic imaging. This feature may be the clue to the diagnosis of pulmonary hypertension (7–10). Cardiac features such as right ventricular (RV) dilatation (11), RV hypertrophy and septal flattening (5) add to pulmonary arterial dilatation as predictors of the presence of PH. In acute PE the relative diameter of the right ventricle to left ventricle is used to predict mortality (12, 13). Measurement of right and left ventricular volume ratio may be a superior approach (13). Right and left atrium measurements on computed tomography (CT) are also known to have diagnostic and prognostic value for pulmonary vascular disease (14–18). Manual cardiac and pulmonary measurements are limited by their time-consuming nature (14), human error, observer variability (19, 20), and observer experience leading to potentially inaccurate predictions and less frequent use.

Historically, volumetric measurements have not been performed on CTPA because it is not typically a cardiac gated acquisition, causing significant cardiac motion artefacts. However, following improvements in CT technology, cardiac structures are now captured with increased clarity on CTPA due to the more rapid acquisition of the cardiac and great vessel structures and therefore diagnostic information is available, despite the lack of cardiac gating.

Automated ventricular volume measurement approaches have been developed in CT showing similar accuracy to cardiac magnetic resonance imaging (MRI) (21), and provide added prognostic value in acute pulmonary embolism (19). There is the need to develop methods to automatically measure the cardiac volume, myocardial hypertrophy, and great vessels on CTPA to provide a comprehensive cardiopulmonary assessment. In addition, it is necessary to determine the generalisability of such a method across hospitals and CT systems (22).

Artificial intelligence is widely used in cardiothoracic applications with utilisation in different diseases for a variety of computer vision tasks (23–26). Semantic segmentation of cardiac chambers is a challenging task which requires the use of automation to minimise the bias effect and to maximise reproducibility (27, 28). Deep learning (DL) has been used successfully in semantic segmentation tasks with high performance in supervised cardiac segmentation (29) including multiple cardiovascular structures (30). The main limitations of the deep learning approaches are the lack of model generalizability across different domains, interpretability and explainability of the model, and for supervised approaches, the need of a large amount of manual segmentation (31).

Automatic segmentation of cardiac chambers has the potential to provide unbiased and robust measurements for the diagnosis and assessment of cardiovascular diseases. By using the information from volumetric anatomical models derived from semantic segmentation, human interpretable diagnostic and prognostic models can be developed. Such models have the potential to transform the management of pulmonary vascular disease, allowing earlier diagnosis in rare diseases such as pulmonary hypertension. Thus, automatic segmentation is a crucial step for the robust and unbiased evaluation of CTPA.

The aims of this retrospective study were:

- to measure the interobserver variability for multi-structure cardiac segmentation in CTPA so that any automated segmentation tool can be compared to human performance of three independent observers.
- to develop a deep learning semantic segmentation tool for CTPA four chamber, ventricle myocardium and great vessel structures and to evaluate the performance of the DL method in an internal and external dataset. The external dataset will be used to assess the generalisability of the segmentation tool utilising a dataset from hospitals across England and Wales.
- to evaluate the failure rates in two disease groups; confirmed pulmonary embolism and suspected pulmonary hypertension to establish the generalisability of the model to different pathologies.
- to investigate the correlations between cardiac structure volumes and invasive haemodynamic measurements for DL and human segmentations.

## Materials and methods

In this study we develop and test a deep learning multi-structure semantic segmentation model which segments the four chambers, myocardium, and great vessels on CTPA scans. An interobserver comparison study was conducted with three observers to measure the accuracy for multi-structure cardiac segmentation in order to contextualise the performance of the deep learning multi-structure segmentation models.

**Figure 1:**
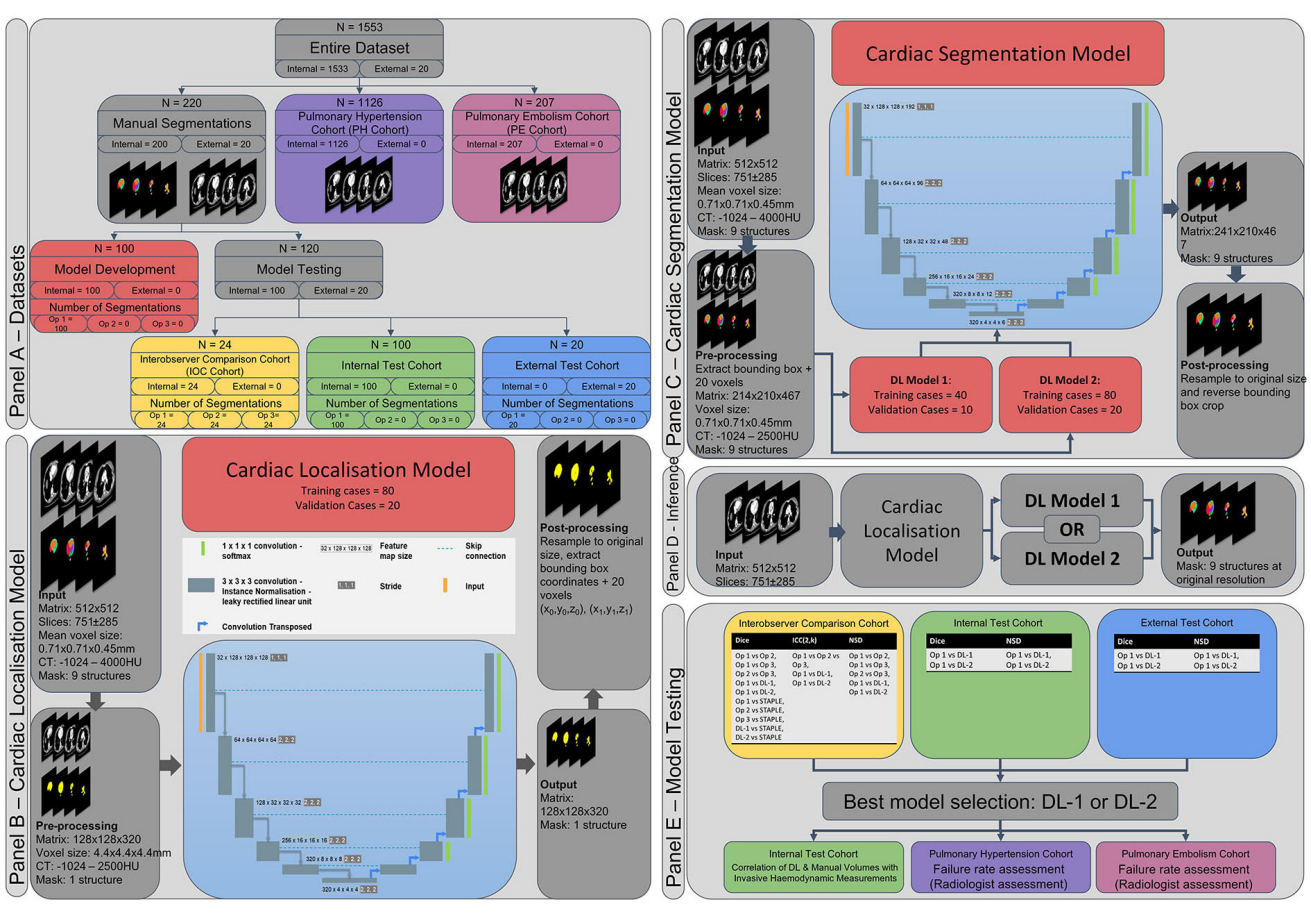
Methodology. **(A)** Datasets used within this study. **(B)** Cardiac localisation deep learning model. **(C)** Cardiac segmentation deep learning model. **(D)** Inference pipeline. **(E)** Model testing strategy. Ob 1, 2, 3, observer 1, 2, 3; DL-1, deep learning model 1; DL-2, deep learning model 2; Dice, dice similarity score, ICC, intraclass correlation coefficient; NSD, normalised surface distance (Surface Dice Score).

For the deep learning segmentation, a two stage, cascade approach is used; firstly, a low-resolution model is trained to localise and extract the cardiac structures within the CTPA scan (Cardiac Localisation Model—Figure 1B), secondly a high-resolution model is trained to segment a multi-structure cardiac model on the extracted cardiac structures (Cardiac Segmentation Model—Figure 1C). For the segmentation, two different models were trained and compared; model 1 (DL-1) and model 2 (DL-2) which were trained with 50 and 100 patients respectively.

The best performing deep learning model was selected for further analysis. Volumetric parameters from human segmentation and segmentations from the best performing model were correlated with invasive haemodynamic pressure measurements in a cohort of 100 PH patients. Segmentation failure rates were measured in a large cohort of 1,333 patients with a variety of cardiovascular disease.

Figure 1 provides an overview of the methodology used in this study; Figures 1A–E show the patient populations and respective cohorts, the cardiac localisation model, the multi-structure cardiac segmentation model, the inference pipeline and the testing methodology respectively.

### Patient populations and datasets

This was a GDPR compliant retrospective study based on 1,553 patients selected from the ASPIRE registry of patients with suspected pulmonary hypertension (*n* = 1346) and patients selected from a local registry of patients with confirmed pulmonary embolism (*n* = 207). Research ethics committee approval for retrospective analysis with waiver of informed consent was obtained for PH patients (ASPIRE, ref: c06/Q2308/8) and PE patients (ref: 17/YH/0142). We followed the CLAIM (checklist for artificial intelligence in medical imaging) (32) checklist for presenting this research.

The selected patients were split into different cohorts used to train and test a deep-learning model. An internal cohort (*n* = 200) and an external cohort (*n* = 20) of patients with suspected PH referred to a tertiary referral centre were identified from the ASPIRE registry. Patients had heterogeneous underlying conditions: lung disease, left heart disease, pulmonary thromboembolic disease, pulmonary arterial hypertension, and a group of patients found to not have pulmonary hypertension following right heart catheterisation. The internal cohort was used for training, validating and testing the deep learning models. The internal test cohort (*n* = 100) was used for correlating volumetric measurements with invasive haemodynamics, with a subset of the internal test patients used for the interobserver comparison (*n* = 24). The external cohort was used for testing the model. Ground truth segmentations in the internal and external cohorts were made by a single consultant cardiothoracic radiologist observer (AJS). Three consultant cardiothoracic radiologists, observer 1 (AJS), 2 (KK), and 3 (CJ), with 12, 5 and 15 years' experience respectively segmented the cardiac structures on the patients in the interobserver comparison cohort.

Failure rates of the segmentation models were tested in a large group of patients with a variety of pulmonary vascular diseases, a suspected PH Cohort (*n* = 1,126) and a confirmed PE Cohort (*n* = 207). See Figure 1A for details of the patient cohorts and Table 1 for the patient demographics.

**Table 1:** Demographics, diagnosis and scanner type of the cohorts utilised.

	**Train DL-1**	**Validation DL-1**	**Train DL-2**	**Validation DL-2**	**Internal test cohort**	**External test cohort**	**PE cohort**	**PH cohort**
Total	40	10	80	20	100	20	207	1,126
Age, years ± SD	65.7 ± 13.6	56.9 ± 15.2	66.2 ± 12.1	58.2 ± 11.9	63.8 ± 13.9	62.7 ± 16.0	64.2 ± 17.0	64.6 ± 12.8
Age range, years	26–81	32–75	26–87	32–75	20–86	24–88	22–95	18–90
Female, %	60.0	70.0	63.7	60.0	62.0	60.0	57.0	62.3
Ethnicity								
White, %	85	80	83	85	85	90	–^*^	89
Black, %	0	0	4	0	6	0	–	2
Asian, %	8	10	6	5	5	5	–	5
Other, %	0	0	0	5	1	0	–	1
Not stated, %	8	10	8	5	3	5	–	3
Manufacturer								
GE, %	50.0	50.0	50.0	40.0	45.0	40.0	85.0	74.2
Canon, %	50.0	50.0	50.0	60.0	55.0	25.0	13.5	25.7
Philips, %	0.0	0.0	0.0	0.0	0.0	5.0	1.4	0.0
Siemens, %	0.0	0.0	0.0	0.0	0.0	30.0	0.0	0.2
Pulmonary hypertension diagnosis								
Not PH, %	17.5	40.0	17.5	25.0	12.0	10.0	0.0	13.6
Pulmonary hypertension with lung disease, %	10.0	20.0	17.5	25.0	15.0	15.0	0.0	19.2
Pulmonary arterial hypertension, %	27.5	20.0	16.3	20.0	19.0	15.0	0.0	21.0
Pulmonary hypertension with left heart disease, %	25.0	20.0	25.0	10.0	22.0	20.0	0.0	16.8
Chronic thromboembolic pulmonary hypertension, %	20.0	0.0	23.8	10.0	25.0	30.0	0.0	26.2
Unclear/unknown, %	0.0	0.0	0.0	10.0	7.0	10.0	0.0	3.0
Pulmonary embolism Positive, %	0.0	0.0	0.0	0.0	0.0	0.0	100	0.0
Invasive haemodynamic measurement available, %	100.0	100.0	98.8	100.0	100.0	75.0	0.0^**^	98.8
Mean PA pressure, mmHg ± SD	39 ± 14	41 ± 21	39 ± 13	42 ± 17	42 ± 14	47 ± 14	–	39 ± 14
PA pressure range, mmHg	16–71	16–77	14–71	16–77	17–78	23–73	–	8–95

PE, pulmonary embolism; PH, pulmonary hypertension; PA, pulmonary artery.

* Patient ethnicities were not available in the PE cohort.

** Invasive haemodynamics were not performed in the PE cohort.

#### Internal cohort

Patients were selected randomly from suspected PH patients imaged at Sheffield Teaching Hospitals NHS Trust between 2010 and 2018 who had undergone right heart catheter measurements within 48 h of CTPA image acquisition. The cohort consisted of 93 patients imaged on a 64 detector-row CT GE system (Light-Speed; General Electric Medical Systems, Milwaukee, WI) and 107 patients imaged on a 320 detector-row Canon CT system (Aquilion ONE/ViSION edition; Canon Medical Systems Corporation, Otawara, Japan). GE acquisition parameters: 120 kV, 100 mA with auto dose reduction, pitch 1, rotation time 0.5 s, field of view (FOV) 400 × 400mm and slice thickness 0.625 mm. Canon acquisition parameters: kV 120, modulated mA, pitch (standard pitch factor 0.813 and helical pitch 65) rotation time 0.275, FOV 500 L and slice thickness 0.3 mm. Intravenous iodine contrast agents were administered with a dose of 60 ml at a rate of 5 ml/s (agent Omnipaque 300, GE Healthcare, United States). Bolus tracking was used with a region of interest over the pulmonary trunk with a manual trigger. Contiguous slices were acquired during an inspiratory breath hold.

#### External cohort

The external cohort consisted of 20 patients from 12 hospitals across England and Wales acquired between 2011 and 2018 on GE (*n* = 8), Siemens (*n* = 6), Philips (*n* = 1) and Canon (*n* = 5) CT scanners.

#### Pulmonary hypertension cohort (PH cohort)

A cohort of 1,126 patients imaged in two hospitals in England was selected from the ASPIRE registry. Consecutive patients between 2011 and 2019 which had invasive right heart catheterization (RHC) haemodynamics within 48 h of the CT were selected. Patients in the internal cohort were excluded. Patients were imaged on GE (*n* = 835), Canon (*n* = 289), and Siemens (*n* = 2) CT scanners. Patients had heterogeneous underlying conditions similar to the internal cohort.

#### Pulmonary embolism cohort (PE cohort)

A cohort of 207 patients with confirmed PE was randomly selected from two hospitals in England between 2009 and 2017. Cases were acquired on GE (*n* = 176), Canon (*n* = 28), and Philips (*n* = 3) CT Scanners.

### Cardiac segmentation model

Nine structures were manually segmented on the CTPA images for the 220 cases in the internal and external cohorts; the left ventricular (LV) myocardium (LV_myo_), LV endocardial cavity (LV_vol_), right ventricular (RV) myocardium (RV_myo_), RV endocardial cavity (RV_vol_), left atrium (LA), right atrium (RA), proximal pulmonary arteries (PA) and the aorta, which was split into two structures (i) the ascending aorta and aortic arch excluding the supra-aortic vessels (Ao_ascend_), and (ii) the descending aorta (Ao_descend_). The left ventricular structures included the septum and the outflow tract and excluded trabeculation. The right ventricular structures included the outflow tract and excluded the septum and trabeculation. The LA excluded the appendage and pulmonary veins. The RA included the appendage and excluded the inferior and superior vena cava. For the aorta the outer margin was segmented, therefore calcifications and atheroma were included where present.

Ground truth image segmentations were performed using MASS software (Version 2021EXP, Leiden University Medical Center, Leiden, The Netherlands). Segmentation was performed on axial slices with interslice contour interpolation used where appropriate (e.g., along the descending aorta) to speed up the process. Multi-planar reformats were reviewed to achieve consistent contouring between slices. For the 100 patients in the internal test cohort contemporary standard of care manual measurements were made; RV:LV ratio of maximal chamber diameter and pulmonary artery: aorta ratio (PA:Ao) of vessel diameter at the level of the PA bifurcation, both measurements made on axial slices. Independent quality control was performed on all manual segmentations by an experienced medical physicist, errors and omissions were corrected by the original observer.

### Deep learning pipeline

Deep convolutional neural networks using nn-UNet (33) were trained for cardiac localisation (Figure 1B) and cardiac segmentation (Figure 1C). Common pre-processing, data augmentation, and network parameters were used for all networks, further details are provided in Supplementary material E1.

#### Cardiac localisation model

A cardiac localisation model was trained to extract a volume containing the heart and great vessels. The nine manually segmented cardiac structures were merged into a single structure (combined cardiac structure) to be used as a training label. Images and labels were pre-processed by resampling to a 4.4 mm isotropic voxel size and a 128 × 128 × 320 matrix. The localisation model allows the heart to be localised in CT acquisitions of up to 1.4 m in length, such as a chest-abdomen-pelvis acquisition. A patch size of 128 × 128 × 128 and batch size of 5 was used. Training/validation/testing was conducted using the DL Model 2 cohort with 80/20/100 patients respectively. No external validation was conducted.

The resulting segmented cardiac structure was used to extract a rectangular cuboid bounding box containing the structures of interest, a symmetrical margin of 20 voxels was added to the bounding box. The bounding box was used to establish the input image volume for inferences using the DL-1 and DL-2 segmentation models.

#### Cardiac segmentation

Two separate deep learning models were trained for cardiac segmentation to investigate the performance gains from increasing the training population from 50 cases to 100 cases. Images and masks were pre-processed by extracting a bounding box encompassing the entire manual segmentations plus a symmetrical margin of 20 voxels. The extracted volumes were resampled to a mean voxel spacing of 0.71 × 0.71 × 0.45 mm. The processed volume size was 214 × 210 × 467 voxels compared to 512 × 512 × 751 ± 285 for the original images. The two models were trained to segment the nine cardiac structures. A patch size of 128 × 128 × 192 was used. External testing was conducted in 20 cases. Testing was performed once for each algorithm with no iterative development.

#### Cardiac segmentation model 1 (DL-1)

Training/validation/testing was conducted using the DL Model 1 cohort of 40/10/100 patients respectively.

#### Cardiac segmentation model 2 (DL-2)

This was trained identically to DL-1 apart from training/validation/testing was conducted using the DL Model 2 cohort of 80/20/100 patients respectively. This cohort was created by adding additional patients to the training and validation sets from DL-1.

### Statistical analysis

Segmentations were compared using an overlap-based-metric [Dice Similarity Coefficient (DSC)], a boundary-based-metric [Normalised Surface Distance (NSD) (34)] and a property-related-metric to measure volumetric differences between structures [volume intraclass correlation coefficients (ICC)] following the recommendations of (35). ICC estimates and their 95% confidence intervals were calculated using SPSS statistical package (version 27, SPSS Inc, Chicago, IL) based on a single-rater, absolute-agreement, two-way random-effects model, ICC (2, 1). Normalised Surface Distances were evaluated at structure specific thresholds (see Table 2) derived from the 95th percentile of the NSD measured from the three consultant radiologists in the interobserver variability study (34). Non-parametric Wilcoxon signed rank test was used to compare the paired means of samples populations as the DSC and NSD values are not normally distributed. Non-parametric Spearman's rank order correlation coefficients were calculated as the relationship between cardiac segmentation volumes and haemodynamic pressure measurements were non-linear.

**Table 2:** Dice similarity coefficients (DSC) and normalised surface distances (NSD) for the three observers and DL model 2 evaluated in the interobserver comparison cohort (*n* = 24).

**Cardiac structure**	**Metric**	**Ob 1 vs. Ob 2**	**Ob 1 vs. Ob 3**	**Ob 2 vs. Ob 3**	**Ob 1 vs. DL-2**
LV endocardial cavity	Mean DSC (95%CI)	0.883 (0.865–0.902)	0.909 (0.897–0.921)	0.891 (0.873–0.910)	0.902 (0.891–0.912)
	Mean NSD, τ = 4.38 mm (95%CI)	0.964 (0.944–0.985)	0.936 (0.905–0.966)	0.950 (0.925–0.975)	0.949 (0.920–0.978)
LV myocardium	Mean DSC (95%CI)	0.785 (0.759–0.810)	0.801 (0.780–0.822)	0.798 (0.777–0.818)	0.808 (0.784–0.833)
	Mean NSD, τ = 3.56 mm (95%CI)	0.962 (0.949–0.976)	0.940 (0.921–0.959)	0.948 (0.931–0.964)	0.956 (0.936–0.975)
RV endocardial cavity	Mean DSC (95%CI)	0.902 (0.894–0.910)	0.915 (0.908–0.921)	0.910 (0.902–0.918)	0.924 (0.916–0.932)
	Mean NSD, τ = 4.02 mm (95%CI)	0.960 (0.951–0.969)	0.940 (0.927–0.952)	0.951 (0.942–0.960)	0.963 (0.947–0.978)
RV myocardium	Mean DSC (95%CI)	0.482 (0.444–0.520)	0.542 (0.508–0.576)	0.537 (0.501–0.573)	0.594 (0.554–0.634)
	Mean NSD, τ = 6.04 mm (95%CI)	0.959 (0.949–0.969)	0.948 (0.939–0.957)	0.944 (0.933–0.956)	0.964 (0.956–0.973)
Left atrium	Mean DSC (95%CI)	0.867 (0.851–0.884)	0.888 (0.871–0.904)	0.896 (0.881–0.910)	0.897 (0.874–0.919)
	Mean NSD, τ = 5.20 mm (95%CI)	0.955 (0.939–0.971)	0.929 (0.912–0.946)	0.967 (0.957–0.977)	0.956 (0.924–0.989)
Right atrium	Mean DSC (95%CI)	0.875 (0.859–0.891)	0.892 (0.883–0.902)	0.876 (0.859–0.894)	0.897 (0.878–0.915)
	Mean NSD, τ = 6.70 mm (95%CI)	0.973 (0.961–0.985)	0.941 (0.925–0.958)	0.942 (0.921–0.963)	0.958 (0.924–0.991)
Ascending aorta	Mean DSC (95%CI)	0.901 (0.893–0.909)	0.932 (0.928–0.936)	0.916 (0.906–0.927)	0.924 (0.919–0.930)
	Mean NSD, τ = 2.51 mm (95%CI)	0.969 (0.960–0.978)	0.936 (0.924–0.949)	0.950 (0.936–0.963)	0.953 (0.938–0.967)
Pulmonary arteries	Mean DSC (95%CI)	0.913 (0.904–0.922)	0.830 (0.819–0.842)	0.838 (0.825–0.851)	0.934 (0.925–0.943)
	Mean NSD, τ = 8.13 mm (95%CI)	0.861 (0.845–0.878)	0.980 (0.971–0.988)	0.885 (0.868–0.901)	0.990 (0.981–0.999)
Descending aorta	Mean DSC (95%CI)	0.879 (0.870–0.888)	0.936 (0.924–0.948)	0.900 (0.890–0.910)	0.910 (0.897–0.923)
	Mean NSD, τ = 2.24 mm (95%CI)	0.976 (0.957–0.994)	0.917 (0.892–0.941)	0.945 (0.926–0.964)	0.937 (0.914–0.959)

The threshold for statistical significance was considered a priori to be *P* < 0.05. Statistical analyses were performed in Python using the SciPy [version 1.8.0 (36)] library with plots generated using matplotlib [version 3.4.2 (37)].

#### Interobserver performance comparison

Interobserver comparison was performed using DSC and NSD for structure segmentations, and ICC for structure volume measurements. DSC and NSD were used to evaluate the DL segmentation models in the inter observer comparison (IOC) cohort against individual observers and against the simultaneous truth and performance level estimation (STAPLE) (38) ground truth from all three observers (Supplementary Figure A). ICC was calculated between DL-2 and observer 1.

#### Evaluation of deep learning pipeline

The two DL segmentation models were evaluated against the ground truth manual segmentation from observer 1 in the internal testing and external cohorts. DSC, NSD, volume ICC and structure volumes were used to evaluate segmentation accuracy and performance. The superior model based on mean DSC and NSD was selected for further analysis in the performance evaluation cohorts. Mean values were compared using paired t-tests. Bland-Altman plots were used to compare volumes between the human ground truth and the superior deep learning model, and to compare DL model 1 and DL model 2.

In the performance evaluation cohorts failure was evaluated visually for each structure by observer 1 who assessed failure on a per structure basis on axial slices; multi planar reformats were available. Failure was assessed on a three-point scale where 0 was an ideal segmentation with no errors, 1 included minor errors but were considered unlikely to change the volume or shape of structures significantly, and 2 included errors considered to significantly change structure volume or shape. A score of 2 was considered a failure.

#### Invasive haemodynamic pressure correlations

Spearman rank correlations (ρ) and bootstrap 95% confidence intervals were calculated between cardiac segmentation parameters and invasive haemodynamics in the internal test cohort for observer 1 and the DL-2 model. The volume of right sided cardiac structures (RV_myo_, RV_vol_, RA and PA) were correlated with mean pulmonary artery pressure (mPAP), and left sided cardiac structures (LV_myo_, LV_vol_, LA, Ao_ascend_ and Ao_descend_) were correlated with the pulmonary artery wedge pressure (PAWP). The Steiger z test (39) was performed to test for differences between dependent variables for the manual and DL correlations.

## Results

Results are presented for the interobserver comparison demonstrating the human performance in multi-structure cardiac segmentation on CTPA images. The deep learning model performance is presented in the internal test and external cohorts. The model trained in 100 cases (DL-2) outperformed the model trained in 50 cases and was selected for further analysis in qualitative analysis and for correlation between segmented structure volumes and invasive haemodynamic measurements.

### Interobserver performance

**Figure 2:**
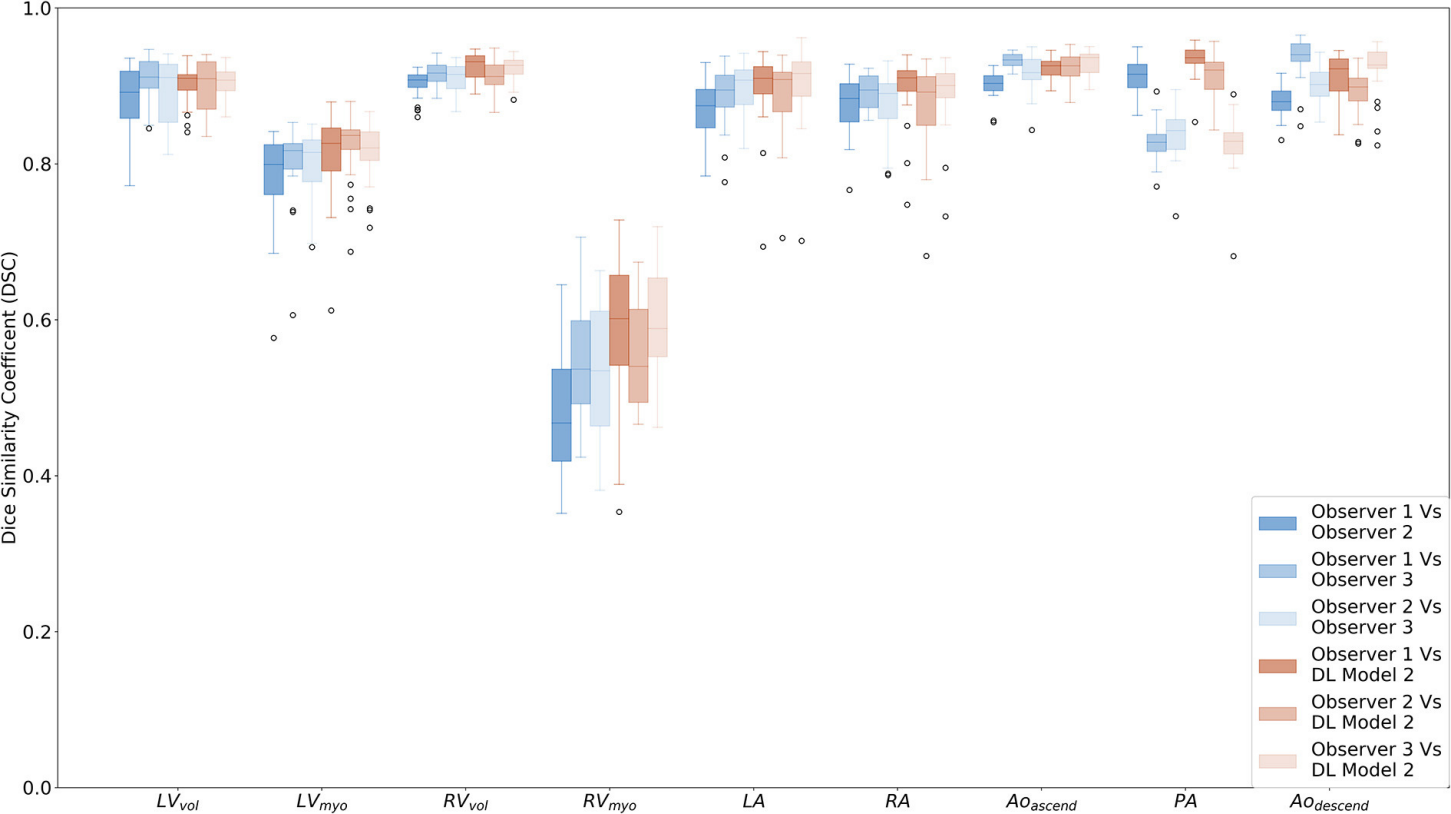
Box plots comparing Dice similarity coefficients (DSC) for the segmented cardiac structures for observers 1 (AJS), 2 (KK) and 3 (CJ) and DL model 2 in the interobserver comparison cohort (*n* = 24). Structures are as follows; LV endocardial cavity (LV_vol_), LV myocardium (LV_myo_), RV endocardial cavity (RV_vol_), RV myocardium (RV_myo_), left atrium (LA), right atrium (RA), ascending aorta and aortic arc (Ao_ascend_), proximal pulmonary arteries (PA), and descending aorta (Ao_descend_).

Mean DSC for the three observers (Table 2; Figure 2) are generally very high, with mean DSC for the LV cavity, RV cavity, LA, RA, ascending aorta, descending aorta, and the pulmonary arteries all within the range 0.830 to 0.936. The DSC for the LV myocardium and the RV myocardium are much lower with mean (range) of 0.795 (0.785–0.801) and 0.520 (0.482–0.542) respectively. Volumetric ICC (2, 1) (Table 3) were excellent and >0.89 for all structures.

**Table 3:** Volume ICC statistics for the three observers and DL model 2 evaluated in the interobserver comparison cohort (*n* = 24).

**Observers**	**Ob 1 and Ob 2 and Ob 3**	**Ob 1 and DL-2**
Metric	Volume, ICC (2, 1) (95%CI)	Volume, ICC (2, 1) (95%CI)
LV endocardial cavity	0.93 (0.74–0.97)	0.93 (0.73–0.97)
LV myocardium	0.91 (0.76–0.97)	0.87 (0.02–0.97)
RV endocardial cavity	0.97 (0.92–0.98)	0.97 (0.93–0.99)
RV myocardium	0.89 (0.79–0.95)	0.92 (0.83–0.97)
Left atrium	0.97 (0.84–0.99)	0.92 (0.82–0.96)
Right atrium	0.98 (0.96–0.99)	0.97 (0.93–0.99)
Ascending aorta	0.96 (0.72–0.99)	0.96 (0.43–0.99)
Pulmonary arteries	0.94 (0.68–0.98)	0.96 (0.88–0.98)
Descending aorta	0.89 (0.50–0.96)	0.96 (0.89–0.98)

Ninety fifth percentile NSD (Table 2; Supplementary Figure B) ranged from 2.2 mm for the descending aorta to 8.1 mm for the pulmonary arteries. The value for the RV cavity is towards the higher end of this range (6.04 mm) indicating the variability of the segmentation for human observers. The measured structure specific 95th percentile values are used to evaluate the NSD performance of the deep learning models. Deep learning model 2 had NSD values of >0.95 in the IOC cohort (Table 2) for all but two of the structures; the LV cavity and the descending aorta which had NSD values of 0.949 and 0.937, respectively.

The mean DSC, and structure specific NSD for DL-2 compared to observer 1 are comparable to the interobserver human results for all structures and the differences between model and human are lower than the inter-observer reproducibility. The ICC (2, 1) between observer 1 and the DL-2 model are >0.87 indicating excellent correlation to human observers.

### Internal and external test performance

**Figure 3:**
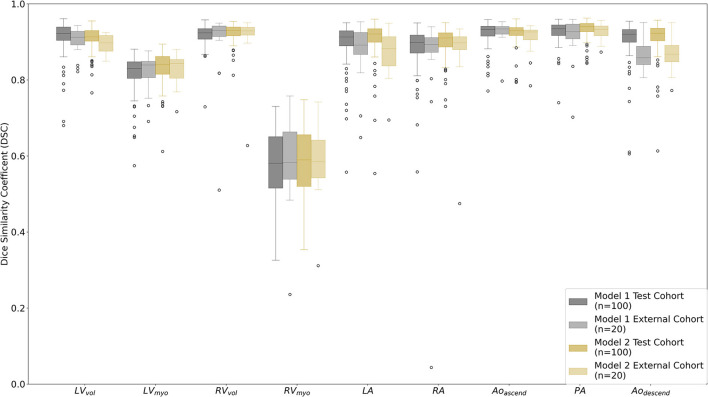
Box plots comparing Dice similarity coefficient (DSC) for the segmented cardiac structures for DL model 1 vs. the manual segmentation observer 1 (AJS) and DL model 2 vs. observer 1 in the test cohort (*n* = 100) and the external cohort (*n* = 20). Structures are as follows; LV endocardial cavity (LV_vol_), LV myocardium (LV_myo_), RV endocardial cavity (RV_vol_), RV myocardium (RV_myo_), left atrium (LA), right atrium (RA), ascending aorta and aortic arc (Ao_ascend_), proximal pulmonary arteries (PA), and descending aorta (Ao_descend_).

**Figure 4:**
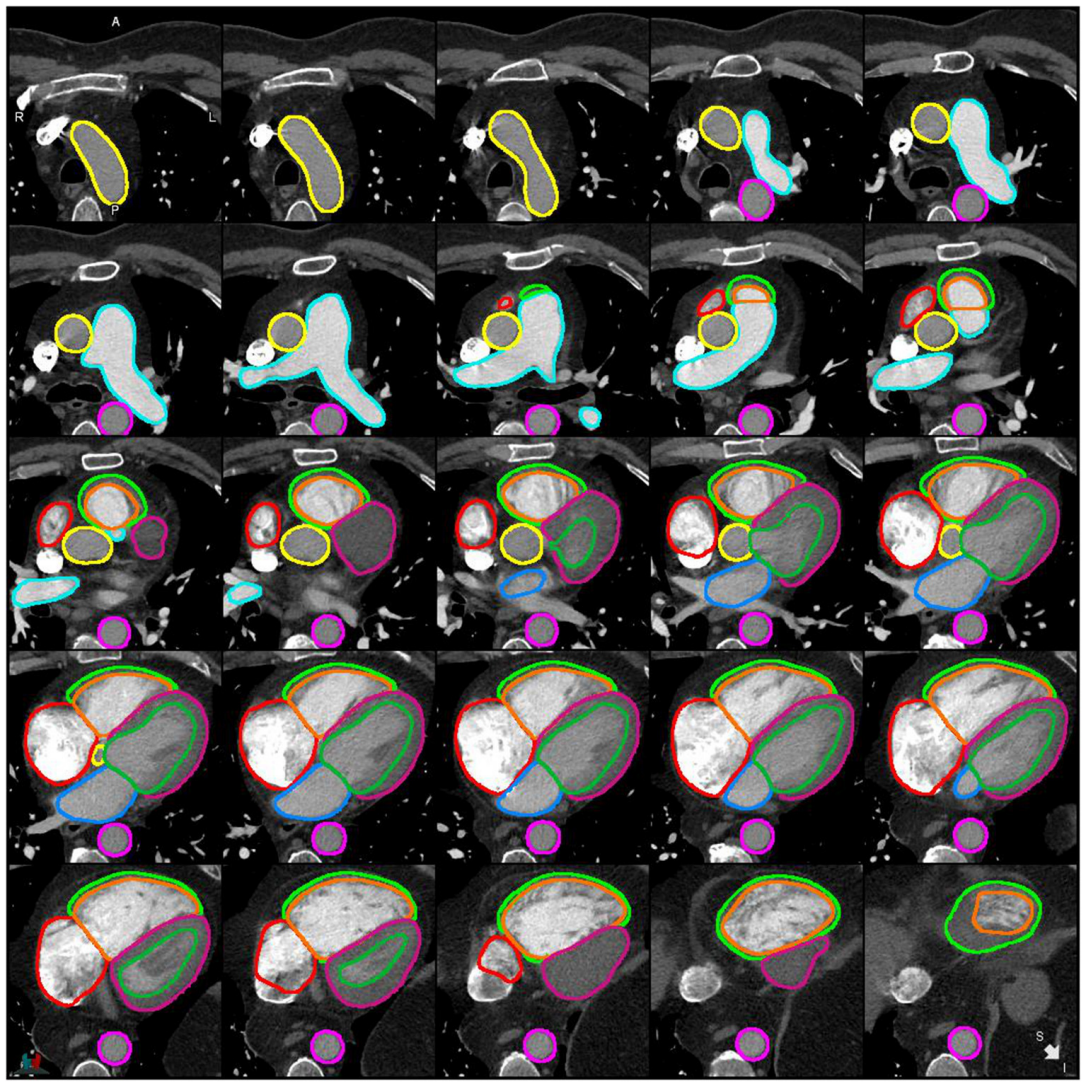
Example of a successful segmentation by DL-2 for a patient with suspected PH in the internal test cohort.

**Figure 5:**
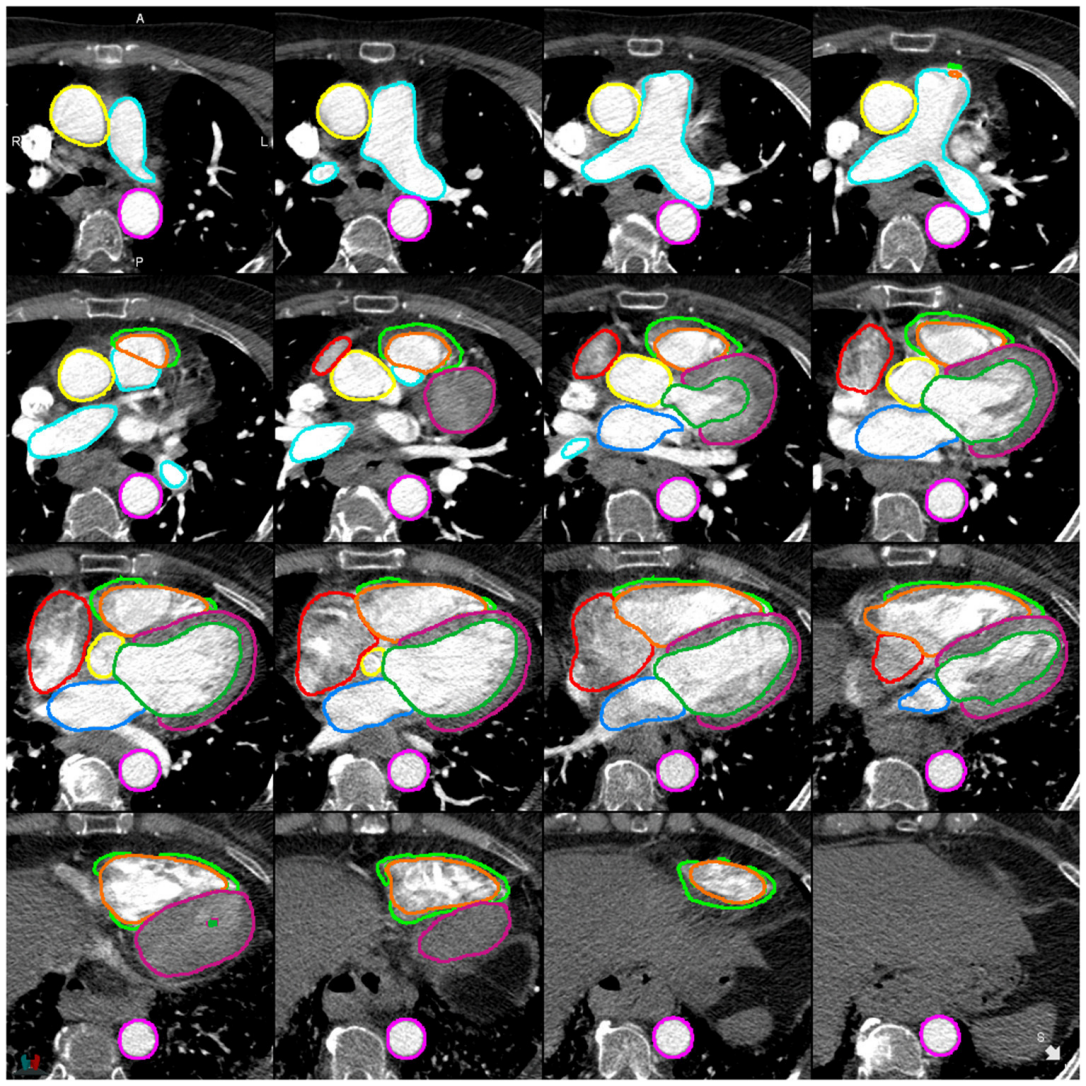
Example of a successful segmentation by DL-2 for a patient with suspected PH in the external test cohort.

Evaluation of DL-1 and DL-2 in the test cohort (*n* = 100) (see Figure 3; Table 4; Supplementary Figure C) demonstrated that the additional fifty training cases used for training DL-2 improved the overall performance of the segmentation. DSC performance was similar for both models in the external cohort, with it being too small a sample (*n* = 20) to reach statistically significant conclusions. Bland-Altman plots for DSC in the test cohort and evaluation cohort are available in the Supplementary Figures D,E. Examples of successful DL-2 segmentation in a suspected PH patients can be found in Figures 4, 5 and Supplementary Videos A (axial) and B (sagittal). The NSD scores in the internal test cohort is marginally improved in DL-2 compared to DL-1 (Table 4).

**Table 4:** Dice similarity coefficients (DSC) and normalised surface distances (NSD) in the internal and external test cohorts for DL model 1 (DL-1) and DL model 2 (DL-2) 2.

**Cohort**	**Internal test cohort (*****n*** = **100)**				**External test cohort (*****n*** = **20)**			
**Cardiac structure**	**Metric**	**DL-1**	**DL-2**	**Difference**	**p-value (t-test)**	**DL-1**	**DL-2**	**Difference**	**p-value (Wilcoxon)**
LV endocardial cavity	Mean DSC (95%CI)	0.911 (0.902–0.920)	0.908 (0.902–0.914)	−0.003	0.42	0.903 (0.886–0.920)	0.894 (0.882–0.906)	−0.009	0.10
	Mean NSD, τ = 4.38 mm (95%CI)	0.957 (0.940–0.974)	0.964 (0.953–0.974)	0.007	0.38	0.948 (0.911–0.986)	0.967 (0.952–0.983)	0.019	1.00
LV myocardium	Mean DSC (95%CI)	0.816 (0.805–0.826)	**0.832 (0.823–0.840)**	0.016	<0.001	0.822 (0.800–0.844)	0.827 (0.808–0.847)	0.006	0.60
	Mean NSD, τ = 3.56 mm (95%CI)	0.952 (0.940–0.964)	**0.963 (0.955–0.972)**	0.012	0.005	0.952 (0.931–0.973)	0.960 (0.947–0.974)	0.009	0.99
RV endocardial cavity	Mean DSC (95%CI)	0.919 (0.914–0.924)	**0.924 (0.920–0.929)**	0.005	0.03	0.899 (0.853–0.946)	0.913 (0.881–0.945)	0.013	0.73
	Mean NSD, τ = 4.02 mm (95%CI)	0.956 (0.944–0.967)	0.965 (0.956–0.973)	0.009	0.07	0.932 (0.883–0.981)	0.950 (0.917–0.983)	0.018	0.25
RV myocardium	Mean DSC (95%CI)	0.577 (0.559–0.595)	**0.584 (0.566–0.603)**	0.007	0.01	0.582 (0.532–0.632)	0.590 (0.546–0.635)	0.008	0.55
	Mean NSD, τ = 6.04 mm (95%CI)	0.954 (0.948–0.960)	**0.958 (0.953–0.964)**	0.004	0.04	0.946 (0.912–0.980)	0.957 (0.935–0.980)	0.011	0.16
Left atrium	Mean DSC (95%CI)	0.897 (0.886–0.909)	**0.907 (0.896–0.917)**	0.010	0.01	0.875 (0.839–0.911)	0.871 (0.842–0.900)	−0.004	0.99
	Mean NSD, τ = 5.20 mm (95%CI)	0.954 (0.937–0.970)	0.962 (0.947–0.977)	0.008	0.12	0.941 (0.890–0.992)	0.912 (0.862–0.962)	−0.029	0.26
Right atrium	Mean DSC (95%CI)	0.886 (0.875–0.897)	**0.898 (0.890–0.906)**	0.012	0.002	0.843 (0.752–0.934)	0.875 (0.829–0.920)	0.032	0.19
	Mean NSD, τ = 6.70 mm (95%CI)	0.944 (0.924–0.963)	**0.967 (0.955–0.979)**	0.024	<0.001	0.901 (0.812–0.989)	0.926 (0.881–0.972)	0.025	0.84
Ascending aorta	Mean DSC (95%CI)	0.924 (0.917–0.930)	0.922 (0.917–0.928)	−0.001	0.62	**0.926 (0.911–0.942)**	0.910 (0.892–0.928)	−0.017	0.02
	Mean NSD, τ = 2.51 mm (95%CI)	0.936 (0.922–0.949)	0.943 (0.931–0.955)	0.007	0.21	0.946 (0.917–0.975)	0.921 (0.889–0.953)	−0.025	0.14
Pulmonary arteries	Mean DSC (95%CI)	0.927 (0.922–0.933)	**0.933 (0.928–0.937)**	0.005	0.01	0.914 (0.887–0.940)	0.927 (0.916–0.937)	0.013	0.08
	Mean NSD, τ = 8.13 mm (95%CI)	0.980 (0.973–0.988)	**0.987 (0.982–0.992)**	0.007	0.003	0.958 (0.912–1.005)	0.968 (0.945–0.990)	0.009	0.62
Descending aorta	Mean DSC (95%CI)	0.906 (0.895–0.917)	0.910 (0.901–0.919)	0.004	0.16	0.866 (0.849–0.884)	0.868 (0.848–0.887)	0.001	0.50
	Mean NSD, τ = 2.24 mm (95%CI)	0.914 (0.898–0.929)	**0.928 (0.914–0.942)**	0.014	0.03	0.876 (0.852–0.900)	0.861 (0.827–0.896)	−0.015	0.65

The bold values indicate the significant values at *p* < 0.05.

**Figure 6:**
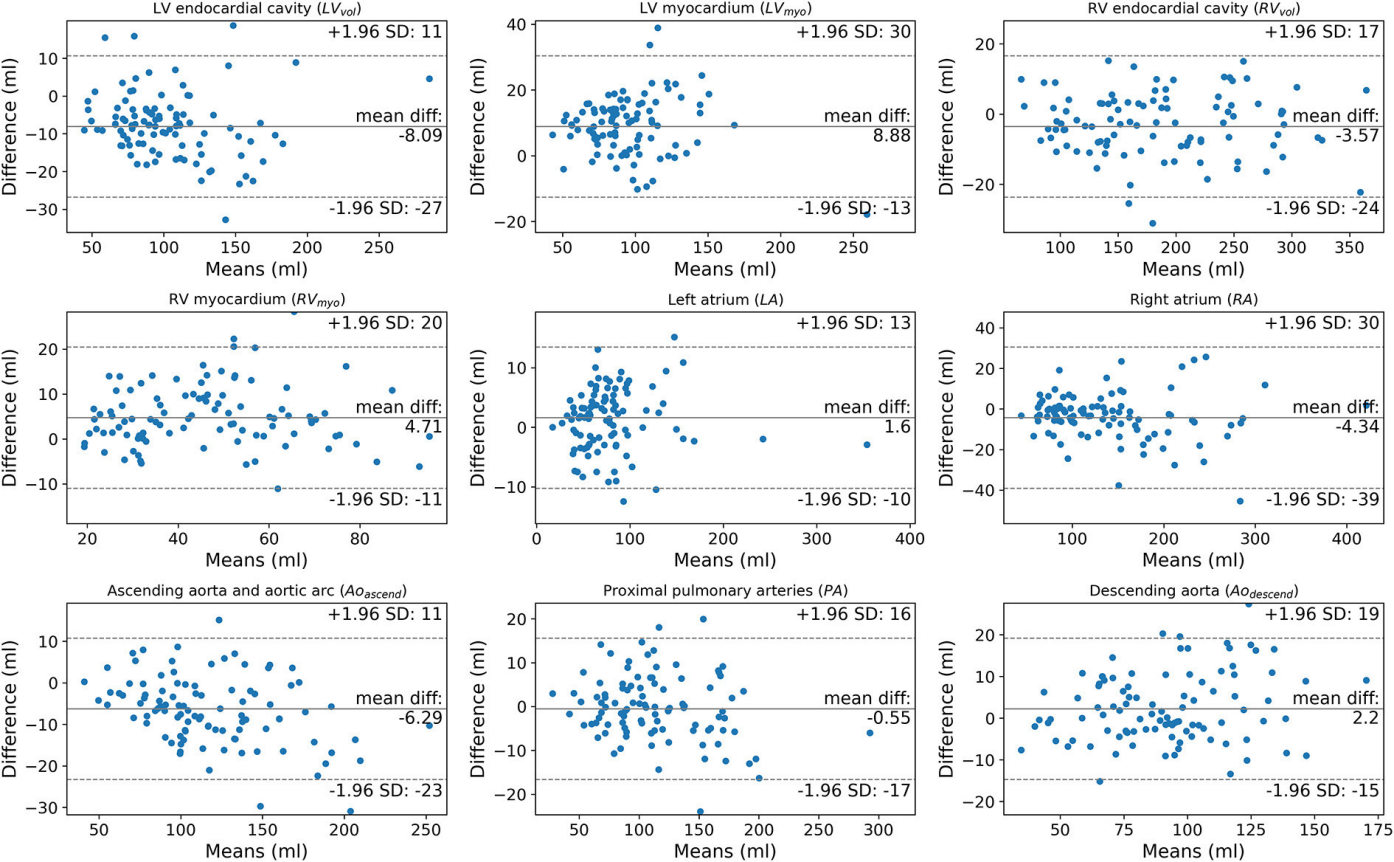
Bland-Altman plots comparing manually segmented structure volumes by observer 1 (AJS) against DL model 2 in the test cohort (*n* = 100).

**Figure 7:**
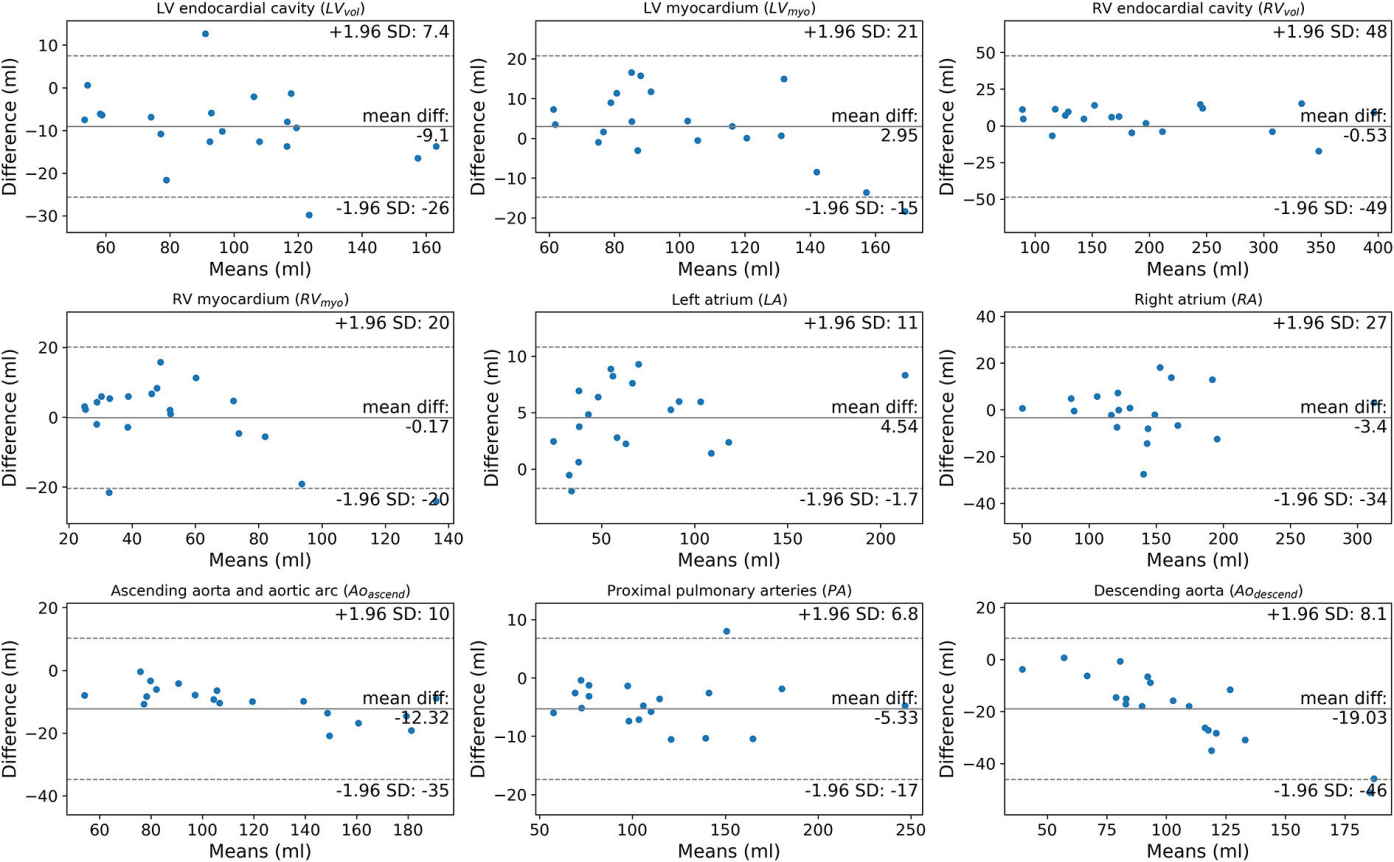
Bland-Altman plots comparing manually segmented structure volumes by observer 1 (AJS) against DL model 2 in the external cohort (*n* = 20).

Volume measurements (Table 5; Figures 6, 7) demonstrate statistically significant differences for human derived volume compared to the DL-2 in both the test cohorts. In the internal test cohort, the mean LV endocardial volume has decreased by 8 ml whereas LV myocardial volume has increased by 9 ml when compared to the manual measurement. This pattern is also seen in the RV myocardial and endocardial volumes but to a lesser extent, suggesting that the DL model is over segmenting thin-walled structures and transferring volume to the myocardium from the endocardial volume as compared to manual segmentation.

**Table 5:** Volumes of segmented cardiac structures for manual (observer 1) and DL model 2 segmentations in the internal and external test cohorts.

**Cohort**	**Internal test cohort (*****n*** = **100)**				**External test cohort (*****n*** = **20)**			
**Metric**	**Mean manual volume, ml**	**Mean DL-2 volume, ml**	**Difference, ml (%)**	**p-value (t-test)**	**Mean manual volume, ml**	**Mean DL-2 volume, ml**	**Difference, ml (%)**	***p*-value (*t*-test)**
LV endocardial cavity	107.75 ± 39.45	99.66 ± 37.45	−8.09 (−7.51)	<0.001	102.37 ± 32.23	93.27 ± 29.30	−9.10 (−8.89)	<0.001
LV myocardium	93.35 ± 36.53	102.22 ± 33.99	8.88 (9.51)	<0.001	100.87 ± 33.13	103.82 ± 27.55	2.95 (2.93)	0.17
RV endocardial cavity	188.34 ± 70.25	184.78 ± 69.22	−3.57 (−1.89)	<0.001	195.92 ± 87.55	195.39 ± 89.39	−0.53 (−0.27)	0.93
RV myocardium	44.48 ± 19.31	49.19 ± 18.44	4.71 (10.60)	<0.001	52.37 ± 30.33	52.20 ± 24.73	−0.17 (−0.33)	0.94
Left atrium	81.66 ± 52.85	83.26 ± 54.71	1.60 (1.95)	0.01	67.04 ± 41.92	71.58 ± 42.93	4.54 (6.77)	<0.001
Right atrium	145.50 ± 74.50	141.16 ± 70.49	−4.34 (−2.98)	0.02	139.34 ± 53.28	135.94 ± 57.51	−3.40 (−2.44)	0.35
Ascending aorta	120.24 ± 42.31	113.95 ± 38.88	−6.29 (−5.23)	<0.001	122.20 ± 41.03	109.88 ± 38.54	−12.32 (−10.08)	<0.001
Pulmonary arteries	116.28 ± 49.26	115.73 ± 46.35	−0.55 (−0.47)	0.50	119.52 ± 45.20	114.19 ± 44.52	−5.33 (−4.46)	0.001
Descending aorta	90.80 ± 27.63	93.00 ± 28.52	2.20 (2.42)	0.01	113.62 ± 42.41	94.59 ± 30.09	−19.03 (−16.75)	<0.001

### Qualitative segmentation performance

DL-2 was assessed in a large cohort (*n* = 1,333) of PH and PE patients; there were 50, 30, and 21 patients with >1, >2 and >3 structures failing, respectively. Overall, there were a total of 148 (1.2%) structures that failed. Failure rates for >1 structures failing (Table 6) in the two cohorts were similar with 3.8 and 3.4% for PH and PE, respectively. The LV myocardium had the highest failure rate which was 2.3 and 1.9% in PH and PE, respectively. Failure of the other structures was generally in the range of 1–1.5%, except for the descending aorta which was 0.4 and 0% in PH and PE, respectively. Radiologists reviewed CT images to identify potential explanations for segmentation failures. Failures were predominantly associated with low or no contrast in one or more chambers of the heart which accounted for 40% of failures. Pericardial effusion and chamber dilatation accounted for 24 and 18% of failures, respectively. The remaining failures were assessed to have been caused by large hiatus hernia, artefacts due to pacemaker/pacemaker leads, severe congenital abnormalities, thoracic deformity, tumour and LV hypertrophy, 4% of failures were associated with image acquisition artefacts. Failure rates were 3.15 and 5.67% for GE and Canon scanners respectively, *p* = 0.07.

**Table 6:** Qualitative failure rates for DL model 2 in the pulmonary embolism (PE), and pulmonary hypertension (PH) cohorts.

**Cohort**	**PH cohort (*n* = 1,126) failure rate (%)**	**PE cohort (*n* = 207) failure rate (%)**	***p*-value (chi squared)**
LV endocardial cavity	1.42	1.45	1
LV myocardium	2.31	1.93	0.94
RV endocardial cavity	1.51	1.45	1
RV myocardium	1.60	1.45	1
Left atrium	0.62	0.48	1
Right atrium	1.60	0.48	0.35
Ascending aorta	0.89	0.97	1
Pulmonary arteries	0.98	1.45	0.81
Descending aorta	0.44	0.00	0.73
Any structure	3.82	3.38	0.92

### Correlations with invasive haemodynamics

Table 7 shows the correlations between mPAP for right sided cardiac structures and PAWP for left sided cardiac structures. The RV myocardial volume had the strongest correlation to mPAP with Spearman rank correlation coefficient (ρ) = 0.70 for DL-2. The LA volume had the strongest correlation to PAWP with ρ = 0.57. The DL-2 correlation was similar to manual segmentation for all structures, with significantly higher correlations for RV_vol_ (0.57 vs. 0.54, *p* = 0.03) and for LV_myo_ (0.34 vs. 0.24, *p* = 0.003).

**Table 7:** Correlation between cardiac structure volumes and invasive haemodynamic measurements for human (observer 1) vs. DL model 2 in the internal test cohort (*n* = 100).

**CTPA parameter**	**Corresponding RHC parameter**	**Human (ρ value)**	**DL model 2 (ρ value)**	***p*-value**
RV endocardial volume	mPAP	0.54 (0.37–0.67)	0.57 (0.41–0.69)	0.03
RV myocardial volume	mPAP	0.68 (0.56–0.77)	0.70 (0.57–0.78)	0.52
Right atrial volume	mPAP	0.41 (0.24–0.55)	0.41 (0.24–0.56)	0.76
Pulmonary arterial volume	mPAP	0.49 (0.32–0.63)	0.51 (0.34–0.64)	0.24
PA:Ao diameter ratio	mPAP	0.50 (0.31–0.65)	0.37 (0.15–0.55)	0.04
RV:LV diameter ratio	mPAP	0.46 (0.27 – 0.61)	0.45 (0.25 – 0.61)	0.83
LV endocardial volume	PAWP	0.23 (0.00–0.43)	0.26 (0.05–0.46)	0.16
LV myocardial volume	PAWP	0.24 (0.03–0.43)	0.34 (0.13–0.52)	0.003
Left atrial volume	PAWP	0.57 (0.39–0.70)	0.57 (0.38–0.70)	0.87
Ascending aorta volume	PAWP	0.20 (0.00–0.38)	0.17 (-0.02–0.36)	0.33
Descending aorta volume	PAWP	0.15 (-0.05–0.35)	0.19 (-0.03–0.38)	0.23

PA:Ao, pulmonary artery to aorta diameter ratio at the level of the PA bifurcation; RV:LV, RV to LV ratio for the maximal mid chamber diameter; mPAP, mean pulmonary artery pressure; PAWP, pulmonary arterial wedge pressure; ρ, Spearman's correlation coefficient. Values in parentheses are 95% CI.

Correlation is higher between mPAP and the DL volume measurement RV_myo_ (ρ = 0.70) compared to the manual contemporary standard of care measurements PV:Ao (ρ = 0.50, p = 0.03) and RV:LV (ρ = 0.46 p < 0.001). When comparing correlations between manual and DL PA:Ao to mPAP and RV:LV to mPAP the manual PA:Ao correlation was found to be significantly stronger than DL-2, ρ = 0.50 vs. ρ = 0.37, *p* = 0.04, for RV:LV no significant differences were found with ρ = 0.46 and ρ = 0.45 for manual and DL respectively, *p* = 0.83.

## Discussion

In this study we show that a deep learning multi-structure four chamber, myocardium and great vessel CTPA segmentation model trained in a mixed cohort of patients with varied underlying cardiothoracic pathology has high accuracy compared to expert cardiothoracic radiologists. Testing has been performed in an internal cohort of patients from the training institution, an external cohort including patients from multiple different hospitals, CT vendors, and pathologies, and a large cohort of patients with two different pulmonary vascular diseases.

Two DL models were trained, the first (DL-1) using 40 training cases and the second (DL-2) using 80 training cases. Both DL models performed well, with the model trained in twice as many patients having a slight performance advantage with mean DSC and NSD in the test cohort increasing by ~1.0%. This improvement in performance is considered insignificant for the additional effort (~30 mins per case) required to generate the manual segmentations. However, it is noted that with the DL-2 model the number of outliers were reduced compared to DL-1 in both the internal and external test cohorts (Figure 3), this may suggest that in some cases increasing the variety in the training cohort by including different pathologies and demographics may be more important than the total size of the training cohort.

In this study we achieve state-of-the-art performance in CTPA segmentation. Prior studies have achieved DSC of 0.85 (40) and 0.92 (41) for semantic segmentation of the pulmonary arteries compared with 0.93 in this study. For whole heart segmentation we refer to the presented results from the multi-modality whole heart segmentation challenge (42) in which seven cardiac structures were segmented on CT angiography scans. In this challenge the RV myocardium was excluded, and the aorta was segmented as a single structure. This challenge was won with a whole heart segmentation DSC of 0.91, which compares very favourably with the value of 0.90 for this study if the RV myocardium is excluded. The DSC values presented in this study exceed the highest score in the challenge table for all right sided cardiac structures; RV endocardial cavity 0.92 vs. 0.91, RA 0.90 vs. 0.89 and PA 0.93 vs. 0.86.

Prior studies have shown that manually derived measures of all four cardiac chambers have clinical value in suspected and confirmed pulmonary vascular disease. In the present study, all structures segmented by DL-2 have excellent accuracy assessed by DSC apart from the LV myocardium (0.83) and RV myocardium (0.58). The low DSC of the LV and RV myocardial measurements between DL-2 and the cardiothoracic radiology observers was mirrored by poor interobserver variability of the radiologists.

The training data for the LV myocardium and the RV myocardium was the weakest of all the chambers. The problem with a Dice Coefficient is that it becomes quite a crude assessment of precision if the ground truth is noisy. If the algorithm identified structures as belonging to the RV, and these were missed by the manual segmentation, the algorithm would be penalised for identifying a true positive. The DSC in the IOC cohort (Figure 2) are similar between each observer and DL-2 with no statistical differences, apart from the PA where observer 3 was found to be segmenting a smaller region than the other two observers. There is no apparent bias towards observer 1 despite all the training cases being generated by observer 1. The volumetric ICC measured in the IOC cohort were excellent for all observers (>0.89) and DL-2 (>0.87) demonstrating that DSC is not the most appropriate metric for assessing thin-walled structures. For this reason, in this study we have used DSC alongside a boundary-based-metric (NSD) to assess the overall segmentation performance. When evaluated using DSC or NSD the differences between model and human is lower than the inter-observer reproducibility within the IOC cohort. The LV and RV myocardium DL and manual segmentations have lower performance highlighting the challenges of segmenting these structures on CT images.

A great challenge of AI development is achieving generalisability of the model across different CT systems, hospitals and diseases (22) and patient populations. The PE and suspected PH cohorts contain patients with a wide range of pathology, including lung disease, left heart disease, pulmonary emboli and congenital heart disease, however the failure rate was found to be comparable for the PE and suspected PH cohorts suggesting good generalisability across pathologies. This study shows similar accuracy of the DL-2 model in patients in the internal test cohort and the external test cohort scanned at 12 hospitals on 4 different CT systems. The DL model was tested and trained in a predominantly white European population (>85% white for all cohorts) and there were insufficient patients of other ethnicities to do a subgroup analysis to determine any bias.

**Figure 8:**
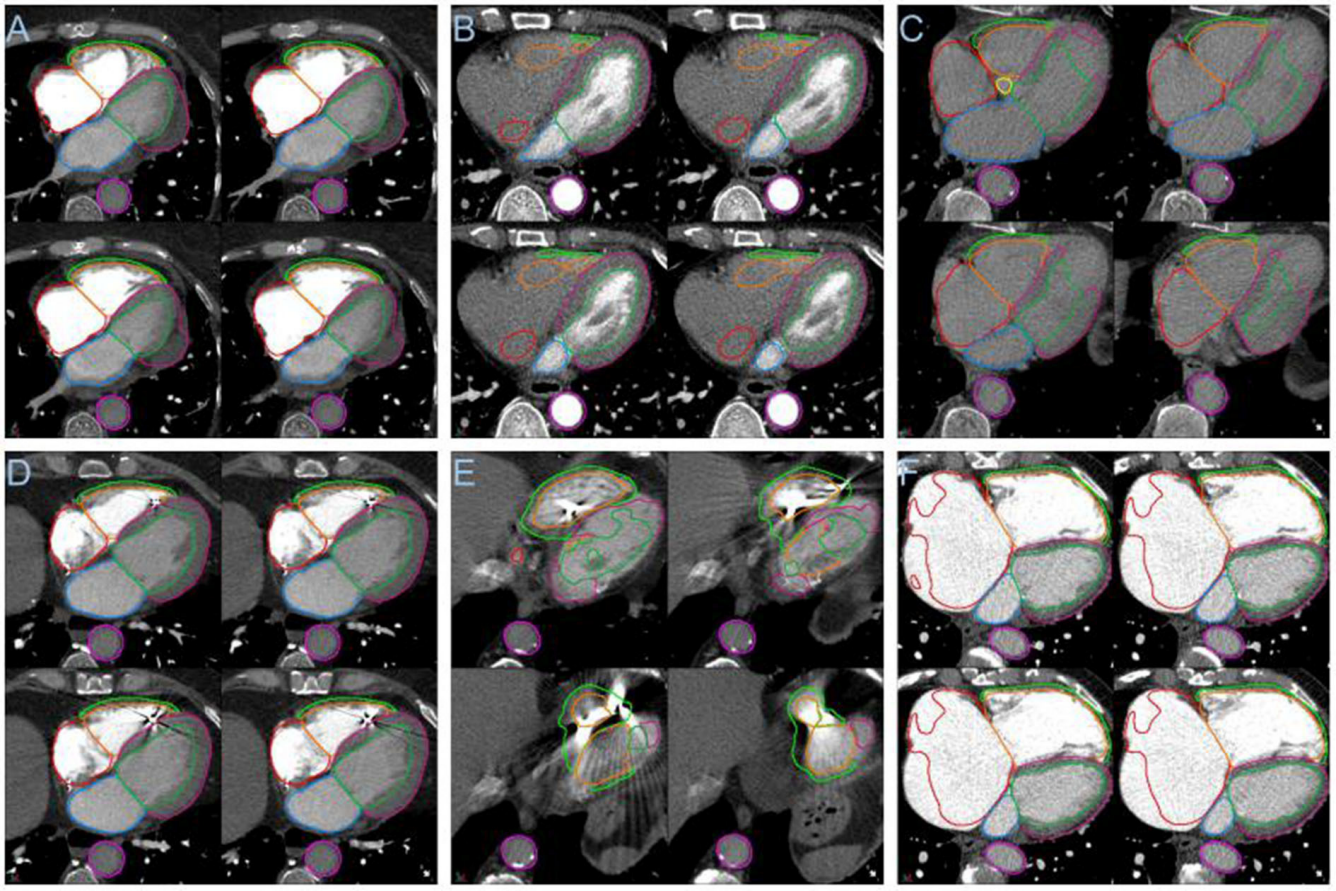
**(A)** Segmentation failure in the LV myocardium in the presence of pericardial effusion. **(B)** Failure of segmentation of right sided cardiac structures with poor right sided contrast opacification. **(C)** Segmentation failure apically with globally poor contrast opacification. **(D)** Example showing success in the presence of an intracardiac device. **(E)** Segmentation failure in the region of an intracardiac device. **(F)** Failure in right atrial segmentation with severe dilatation.

In the large PH and PE cohorts the failure rate was low, <3.9% for failure of any structure. The pathologies causing failures were pericardial effusions see Figure 8A, the poor differentiation of effusion and LV myocardium primarily led to LV epicardial contour failure. Poor contrast opacification was a major cause of failure, see Figures 8B,C. The segmentation performance may be improved by including additional patients with very poor contrast in the training dataset, or alternatively a method for identifying poor opacification prior to automated segmentation may be desirable, as it may not be appropriate to analyse such cases. The model generally performed well in patients with intracardiac devices such as pacemakers, see Figure 8D. Though failures in such cases were identified, see Figure 8E. Local and global chamber dilatation was thought to be the cause for several failures, see Figure 8F, and the addition of more extreme data to the training cohort may improve the performance of the DL model in these cases.

Correlation with mPAP was found to be significantly higher for the DL volume measurement RV_myo_ (ρ = 0.70) compared to the contemporary standard of care measurements PV:Ao (ρ = 0.50, *p* = 0.03) and RV:LV (ρ = 0.46; *p* < 0.001). Despite there being bias between manual and DL volumes for the left and right ventricular structures, the DL volume measurement LV_myo_ shows a significant improvement in the volume correlations with PAWP compared to the manual measurement. All other correlations are comparable between the DL model and manual. For the contemporary standard of care diameter ratio measurements it is interesting to note that the manual correlation for PA:Ao to mPAP is significantly stronger for manual measurements than for the DL measurement, whereas the RV:LV ratio has no significant difference between manual and DL. This may indicate that the method to extract the PA:Ao diameter ratio from the DL segmentations requires refinement to correctly select the level of the PA bifurcation in order to be directly comparable to the manual measurement.

## Limitations and future work

The model developed in this work is specific to ungated CT pulmonary angiography (CTPA), however as ungated CTPA is a very frequent examination, particularly in the emergency department, it is important to have a DL model that works on ungated images. The model has been developed and tested in a predominantly white European population, with a small external test cohort. Future work will seek to address the limitations of this study by testing the DL model in a large cohort of multi-ethnicity patients.

Volumetric measurements are generated in this study, whereas in clinical practise simple diameter measurements tend to be made. This study has highlighted the potential added value of DL volumetric measurements compared to manual diameter measurements in a small cohort. Future work will be to refine the extraction of diameters and ratios and compare with manual approaches used in clinical practise. Further investigation of whether volumetric parameters are of greater clinical significance is required, and the development of diagnostic and prognostic CTPA models for different pulmonary vascular diseases.

## Conclusion

Based on our knowledge, this study presents the first multi-structure four chamber cardiac and great vessel segmentation model that has been developed for CTPA images. We have achieved state of the art accuracy and low failure rates during testing in heterogeneous internal and external cohorts with a wide variety of pulmonary vascular disease. We have used a rigorous testing methodology to evaluate the model and demonstrate that the model is generalisable across different CT vendors and hospitals with differing acquisition protocols. The model has been assessed in different pulmonary vascular diseases with no differences in failure rates identified. The segmented results are highly reproducible compared to multi-structure segmentation performance for expert cardiothoracic radiologists which has been measured in an interobserver comparison study.

Imaging biomarkers based on deep learning volumetric measurements of cardiac structures show strong correlation with invasive haemodynamic measurements and are equal to, or outperform, human volume measurements. Furthermore, the volumetric measurements show superior correlation to invasive haemodynamics than the current standard of care diagnostic measurements (RV:LV and PA:Ao ratio), offering the potential for diagnostic and prognostic models from routine CTPA imaging.

## Data availability statement

The algorithm will be made available on reasonable request for research use. Requests should be sent to the corresponding author MS, michael.sharkey3@nhs.net.

## Ethics statement

The studies involving human participants were reviewed and approved by North Sheffield Ethics Committee. Written informed consent for participation was not required for this study in accordance with the national legislation and the institutional requirements.

## Author contributions

MS, SA, JT, AS, PM, RC, and DK contributed to the database. MS produced the deep learning models. MS, KD, and AS conducted analyses. MS and AS wrote first draft with MM contributing a section. KK, CJ, SR, AS, and DA conducted image analysis. AS, DK, RC, RG, DO'R, PG, JT, and MS contributed to the conception and design of the study. All authors contributed to manuscript revision, read, and approved the submitted version.

## Funding

This work was supported by an NIHR AI Award, AI_AWARD01706. AS was supported by a Wellcome Trust Fellowship Grant 205188/Z/16/Z which provides the open access publication fees for this manuscript. DO'R was supported by the Medical Research Council (MC-A658-5QEB0) and British Heart Foundation Grants (RG/19/6/34387 and RE/18/4/34215).

## Conflict of interest

The authors declare that the research was conducted in the absence of any commercial or financial relationships that could be construed as a potential conflict of interest.

## Publisher's note

All claims expressed in this article are solely those of the authors and do not necessarily represent those of their affiliated organizations, or those of the publisher, the editors and the reviewers. Any product that may be evaluated in this article, or claim that may be made by its manufacturer, is not guaranteed or endorsed by the publisher.
