# Supplementary material for: Fully automatic cardiac four chamber and great vessel segmentation on CT pulmonary angiography using deep learning
Source: Front Cardiovasc Med. 2022 Sep 26;9:983859. doi: 10.3389/fcvm.2022.983859 (PMC9549370; doi:10.3389/fcvm.2022.983859)
Supplement: Supplementary file 1 [file Data_Sheet_1.docx]

# Supplementary Material

## E1. Image pre-processing, data augmentation and model parameters

### Deep learning hyperparameters

CT images contain approximately 4000 times more voxels compared to a cardiac MRI (250 million voxels for a 3D volumetric CT vs. 64 thousand pixels for a 2D slice MRI). This is significantly more demanding on the GPU memory requirements for training a deep learning segmentation model. For a deep learning model to perform well the patch size needs to be greater than, or comparable to, the size of the organ(s) that are being segmented, and the batch size is ideally large so that the gradients converge smoothly. In order to meet these two requirements for large CT images (for a fixed GPU memory) either the image can be down sampled resulting in a loss of information and reduced accuracy of the segmentation due to partial volume effects, or a cropped image centred on the target organ can be extracted as a pre-processing step. For this experiment a combination of the two approaches was used in order to minimise the partial volume effects, first a pre-processing step extracted a volume containing only the heart and great vessels, second a small down sampling step was applied to the extracted volume.

Images were pre-processed by clipping the grayscale to within the range -200 to 1500 HU and normalised by subtracting their mean (293 HU) and dividing by their standard deviation (250 HU) as measured within the combined cardiac structure in the DL-Model 2 training data (n=80). During each training iteration three image and mask patches were extracted from each case by random crops with 50% chance of being centred on a foreground voxel. The following random data augmentations were applied:

- zoom applied between 0.9 and 1.2 with probability of 0.15 (image and mask)
- Gaussian noise added with a standard deviation of 0.01 with probability of 0.15 (image only)
- 3D symmetrical Gaussian smooth with sigma between 0.5 and 1.15 with probability of 0.15 (image only)
- intensity scale between 0.7 and 1.3 with probability of 0.15 (image only)
- per axis flip about each of the x, y and z axes was applied with a probability of 0.5 (image and mask)

Trilinear interpolation was used for the resampling of the images and nearest neighbour interpolation for the mask.

Models were implemented in Python (version 3.8.5, Python Software Foundation, Delaware, United States) using MONAI (version 0.8) (1). Networks had 3 deep supervision (2) layers. A learning rate of 0.01 was used, a SGD-Nesterov optimizer was used with a Dice plus cross entropy loss function. A maximum of 1000 training epochs were used with early stopping. Weights for each model were initialised with random values. Training was conducted using a single 48GB Nvidia A6000 GPU. Inferences require approximately 12GB GPU memory.

### References:

1. MONAI Consortium. MONAI: Medical Open Network for AI. Zenodo: MONAI (2022). doi: 10.5281/ZENODO.4323058
2. Lee C-Y, Xie S, Gallagher P, Zhang Z, Tu Z. Deeply-Supervised Nets. Artificial Intelligence and Statistics. PMLR (2015). p. 562–70 doi:10.48550/arXiv.1409.5185


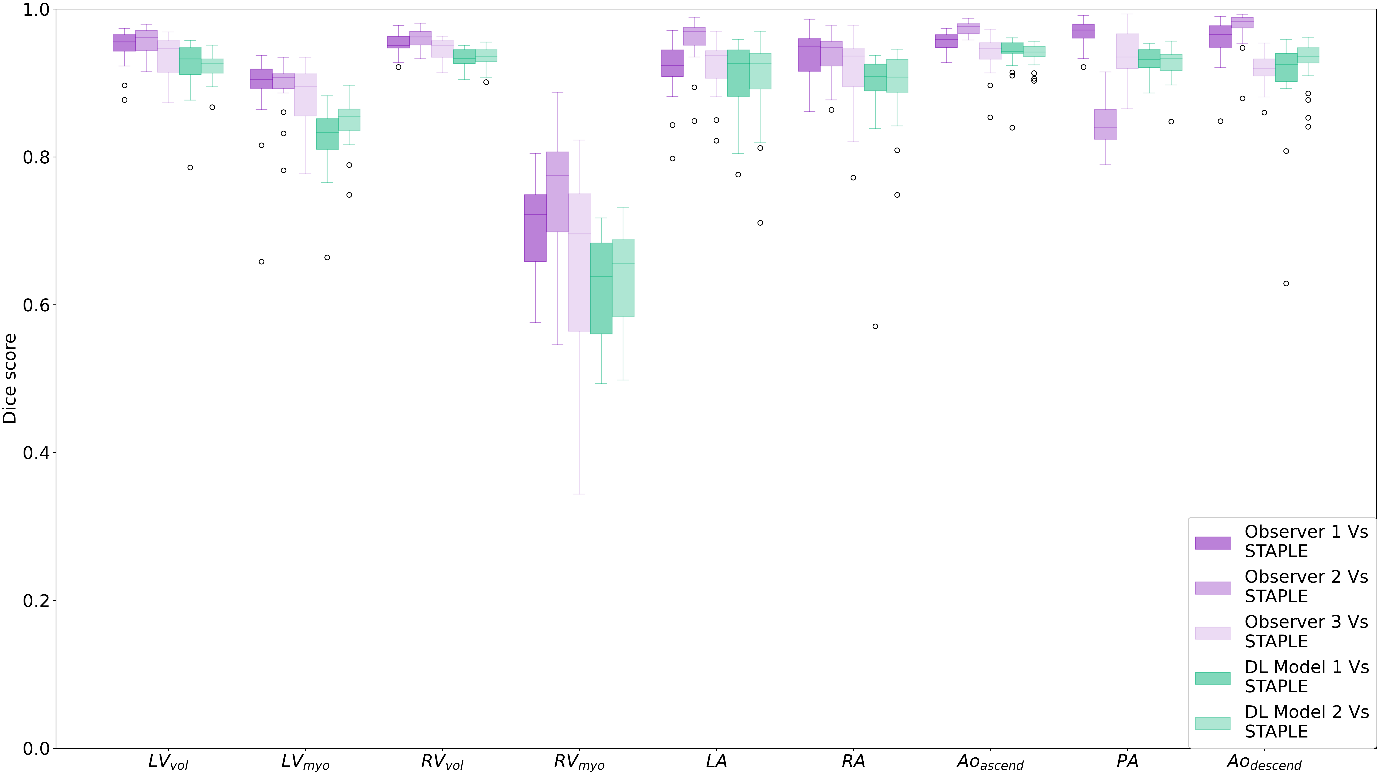


**Supplementary Figure A:** Box plots comparing Dice similarity coefficients (DSC) for the segmented cardiac structures for observers 1-3, and DL-1 and DL-2 versus the STAPLE combined ground truth from observers 1-3 in the interobserver comparison cohort (n=24). Structures are as follows; LV endocardial cavity (LV_vol_), LV myocardium (LV_myo_), RV endocardial cavity (RV_vol_), RV myocardium (RV_myo_), left atrium (LA), right atrium (RA), ascending aorta and aortic arc (Ao_ascend_), proximal pulmonary arteries (PA), and descending aorta (Ao_descend_)


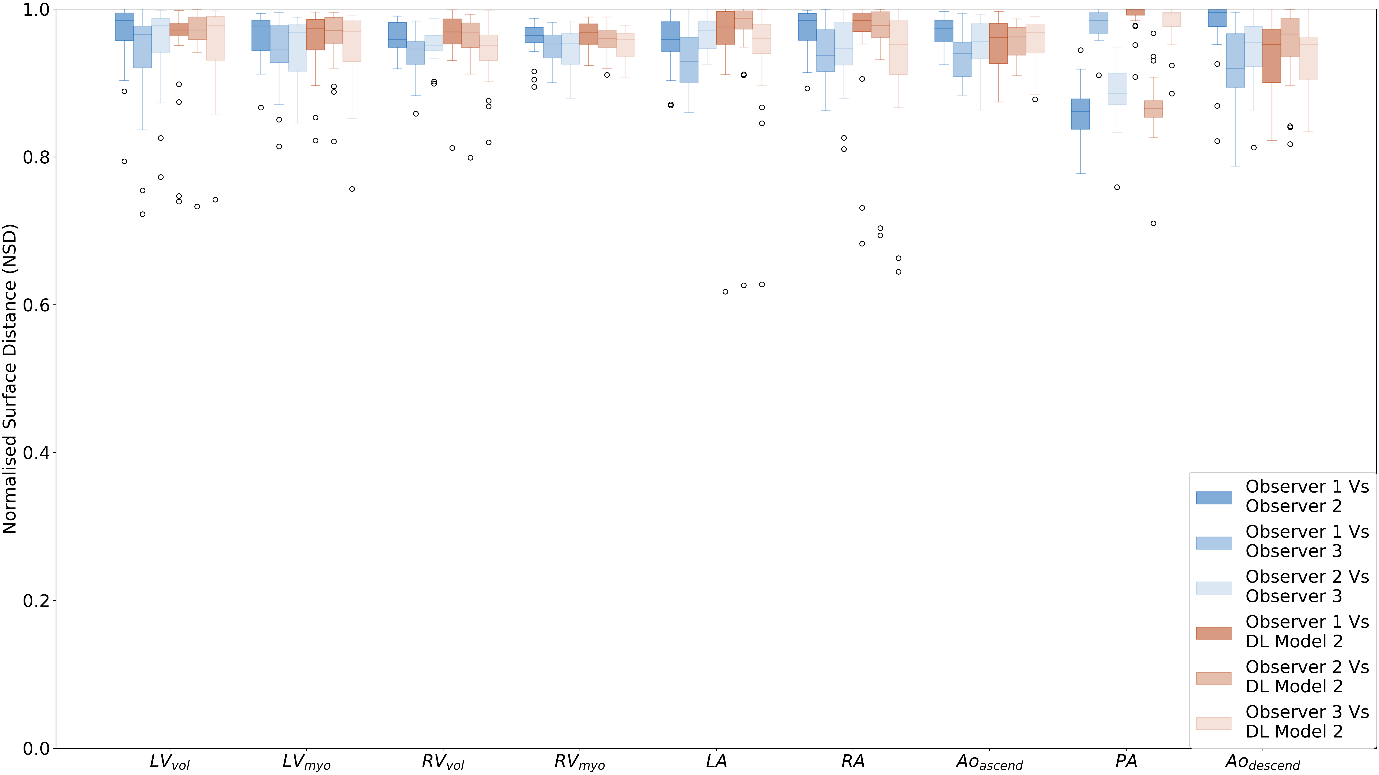


**Supplementary Figure B:** Box plots comparing Normalised Surface Distance (NSD) for the segmented cardiac structures for observers 1 (AJS), 2 (KK) and 3 (CJ) and DL model 2 in the interobserver comparison cohort (n=24). Structures are as follows; LV endocardial cavity (LV_vol_), LV myocardium (LV_myo_), RV endocardial cavity (RV_vol_), RV myocardium (RV_myo_), left atrium (LA), right atrium (RA), ascending aorta and aortic arc (Ao_ascend_), proximal pulmonary arteries (PA), and descending aorta (Ao_descend_)


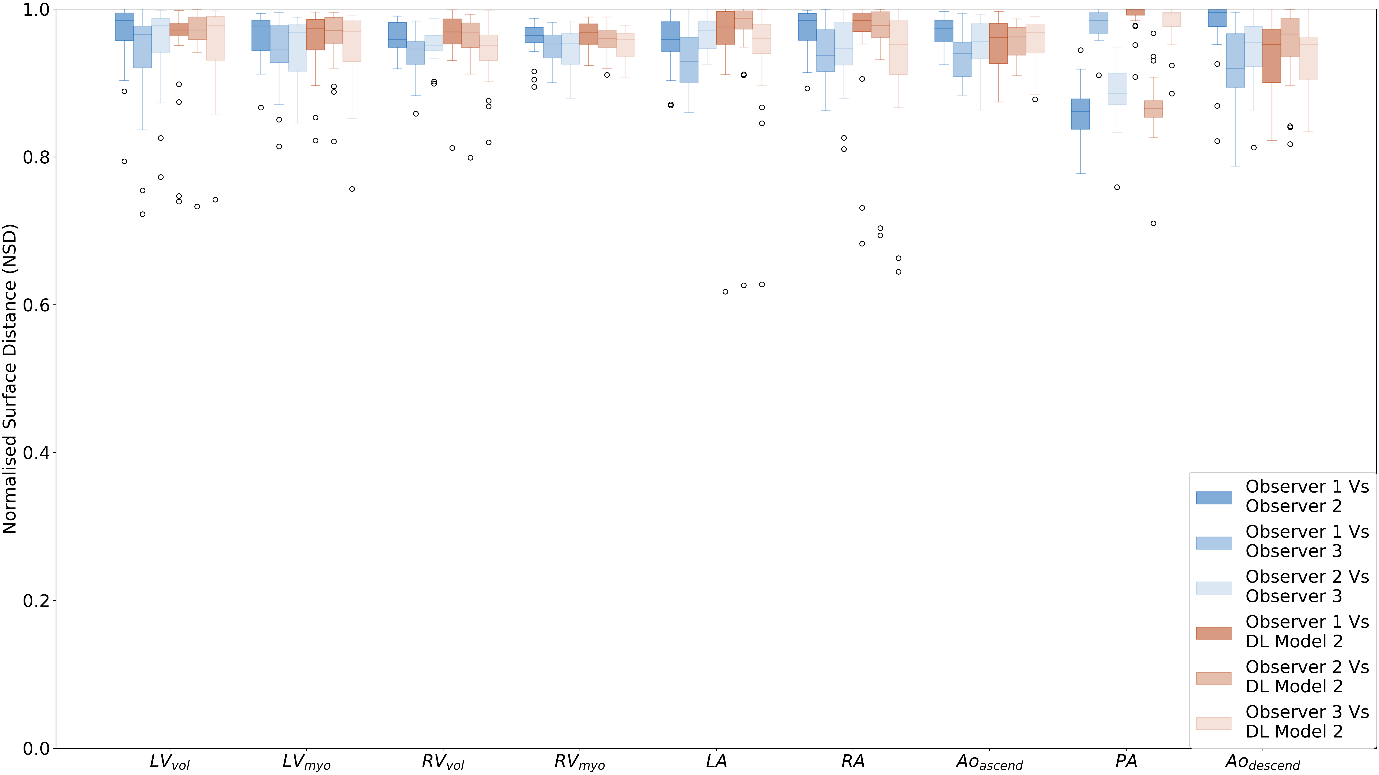


**Supplementary Figure C:** Box plots comparing Normalised Surface Distance (NSD) for the segmented cardiac structures for DL model 1 versus the manual segmentation observer 1 (AJS) and DL model 2 versus observer 1 in the test cohort (n=100) and the external cohort (n=20). Structures are as follows; LV endocardial cavity (LV_vol_), LV myocardium (LV_myo_), RV endocardial cavity (RV_vol_), RV myocardium (RV_myo_), left atrium (LA), right atrium (RA), ascending aorta and aortic arc (Ao_ascend_), proximal pulmonary arteries (PA), and descending aorta (Ao_descend_)


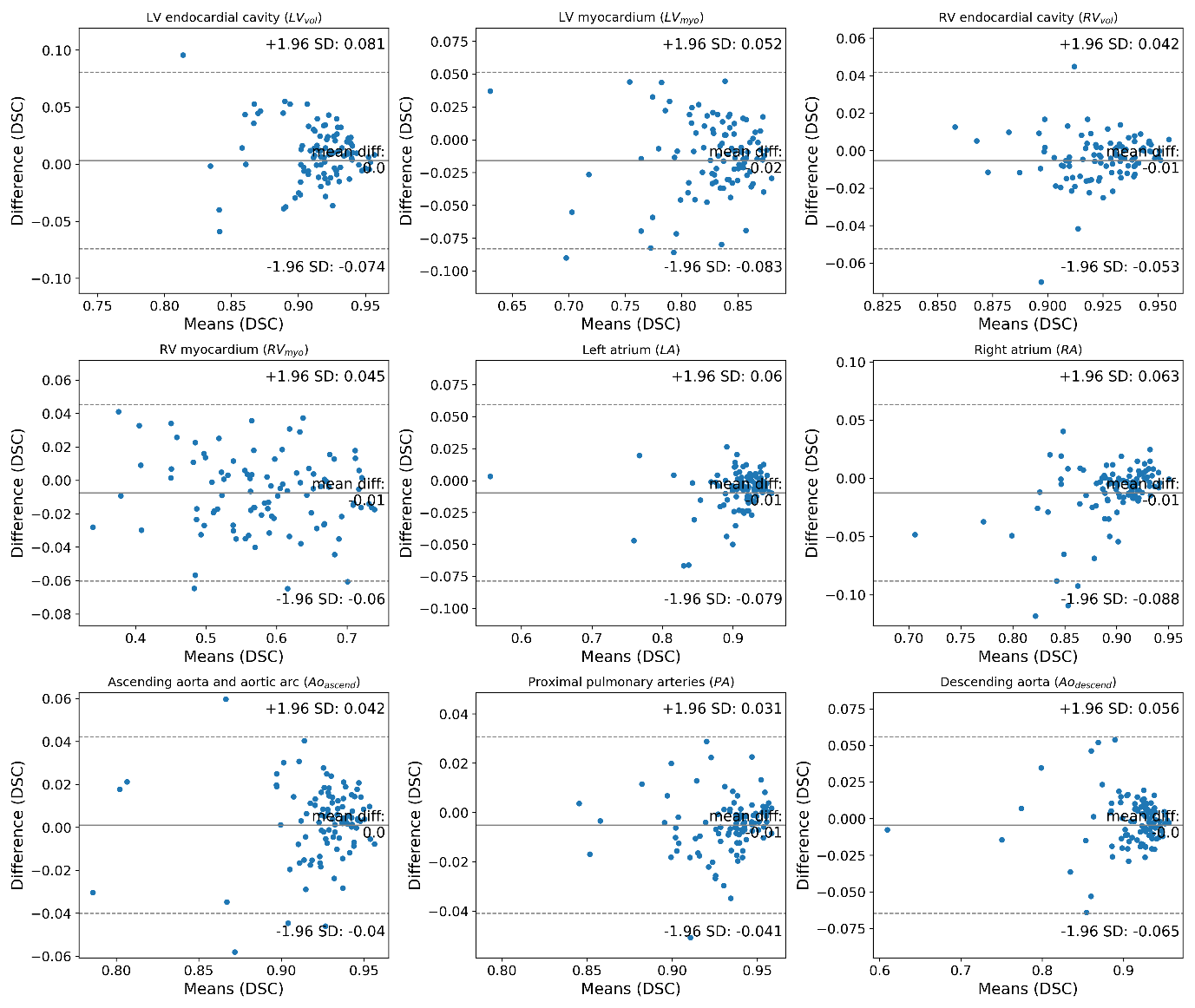


**Supplementary Figure D:** Bland-Altman plots comparing Dice similarity coefficient (DSC) in the test cohort (n=100) for each segmented structure obtained by DL model 1 against DL model 2


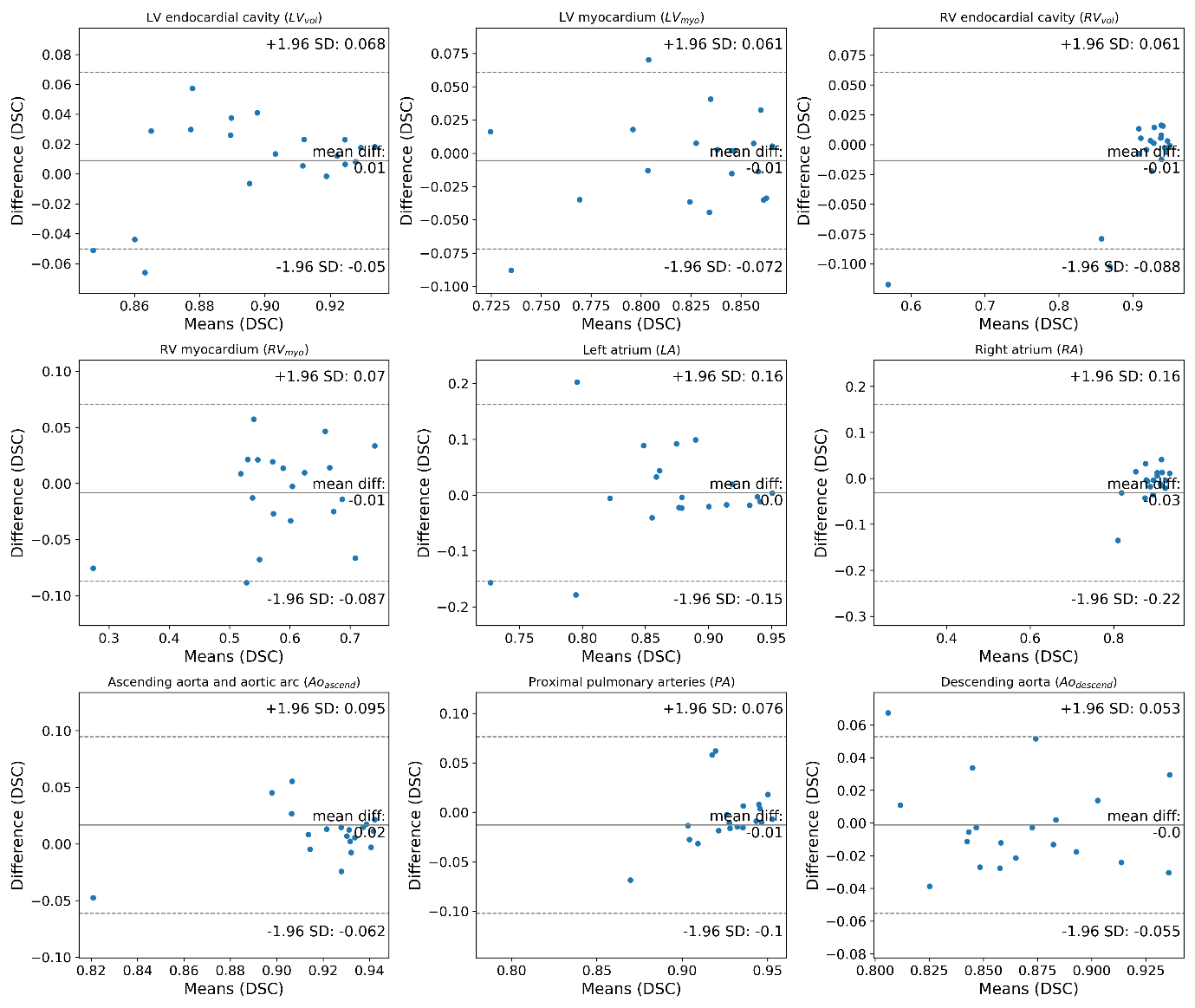


**Supplementary Figure E:** Bland-Altman plots comparing Dice similarity coefficient (DSC) in the external cohort (n=20) for each segmented structure obtained by DL model 1 against DL model 2
